# Comparison of Rift Valley fever virus replication in North American livestock and wildlife cell lines

**DOI:** 10.3389/fmicb.2015.00664

**Published:** 2015-06-30

**Authors:** Natasha N. Gaudreault, Sabarish V. Indran, P. K. Bryant, Juergen A. Richt, William C. Wilson

**Affiliations:** ^1^Arthropod-Borne Animal Diseases Research Unit, Center for Grain and Animal Health Research, Agricultural Research Service, United States Department of AgricultureManhattan, KS, USA; ^2^Diagnostic Medicine and Pathobiology, College of Veterinary Medicine, Kansas State UniversityManhattan, KS, USA

**Keywords:** Rift Valley fever virus, virus replication, permissiveness, livestock diseases, wildlife reservoir

## Abstract

Rift Valley fever virus (RVFV) causes disease outbreaks across Africa and the Arabian Peninsula, resulting in high morbidity and mortality among young domestic livestock, frequent abortions in pregnant animals, and potentially severe or fatal disease in humans. The possibility of RVFV spreading to the United States or other countries worldwide is of significant concern to animal and public health, livestock production, and trade. The mechanism for persistence of RVFV during inter-epidemic periods may be through mosquito transovarial transmission and/or by means of a wildlife reservoir. Field investigations in endemic areas and previous *in vivo* studies have demonstrated that RVFV can infect a wide range of animals, including indigenous wild ruminants of Africa. Yet no predominant wildlife reservoir has been identified, and gaps in our knowledge of RVFV permissive hosts still remain. In North America, domestic goats, sheep, and cattle are susceptible hosts for RVFV and several competent vectors exist. Wild ruminants such as deer might serve as a virus reservoir and given their abundance, wide distribution, and overlap with livestock farms and human populated areas could represent an important risk factor. The objective of this study was to assess a variety of cell lines derived from North American livestock and wildlife for susceptibility and permissiveness to RVFV. Results of this study suggest that RVFV could potentially replicate in native deer species such as white-tailed deer, and possibly a wide range of non-ruminant animals. This work serves to guide and support future animal model studies and risk model assessment regarding this high-consequence zoonotic pathogen.

## Introduction

Rift Valley fever virus (RVFV) belongs to the genus *Phlebovirus* in the family *Bunyaviridae*, and is a vector transmitted pathogen that causes endemic disease across Africa and the Arabian Peninsula (Balkhy and Memish, 2003; Grobbelaar et al., 2011). Periodic outbreaks of the disease coincide with heavy rainfall and flooding conditions which allow for increased breeding of blood-feeding mosquitoes which are the primary vectors for this viral pathogen (Davies et al., 1985; Linthicum et al., 1985; Grobbelaar et al., 2011). RVFV threatens both human and animal health, and has costly economic impact related to livestock production and trade. In affected countries, RVFV causes outbreaks of high morbidity and mortality among young domestic sheep, goats, and cattle, and numerous abortions in pregnant animals (Coetzer, 1977, 1982; Pepin et al., 2010). Humans can also become infected, primarily through contact with infectious animal fluids and tissues. Disease in humans can be mild to severe and in some cases fatal (Pepin et al., 2010; Ikegami and Makino, 2011). Human patients infected with RVF may suffer from a self-limiting, febrile illness, or in more severe cases develop hemorrhagic fever, hepatitis, neurologic disorders, or blindness (Ikegami and Makino, 2011). The human mortality rate varies, but is estimated at 1% of those infected with RVFV. However, fatalities reaching 29% was documented for the RVFV outbreak in east Africa during 2006–2007 (Pepin et al., 2010).

The potential of RVFV spreading to the United States (US) or other countries worldwide, whether by accident or with intent, is of significant concern (Ahmed et al., 2009; Hartley et al., 2011). Several genera of mosquitoes present in the US have been identified as potential competent vectors for this virus (Turell et al., 2008, 2010; Iranpour et al., 2011). Field investigations for seroprevalence in indigenous wildlife of endemic countries and a limited number of animal model studies have demonstrated that RVFV is capable of infecting a wide range of wild animal species (Olive et al., 2012). The major mechanism for persistence of RVFV during inter-epidemic periods is putatively through infected adult mosquitoes passing the virus to their offspring by transovarial transmission (Linthicum et al., 1985), but could also be by means of a wildlife reservoir, as in the case of West Nile virus (Blitvich, 2008). However, to date, no predominant wildlife reservoir has been identified for RVFV, and gaps in our knowledge of permissive hosts still remain, especially concerning the Americas (Hartley et al., 2011).

Wild ruminants in North America, such as deer, could represent an important risk factor given their abundance, wide distribution, and overlap with livestock farms and human populated areas (Kasari et al., 2008; Kakani et al., 2010). Although mosquitoes may have host preferences, they are opportunistic feeders which could facilitate transmission between proximal wildlife, livestock, and human populations (Molaei et al., 2008, 2009a,b). If RVFV were to be introduced into the US and wildlife became infected, it could dramatically impact the establishment and dissemination of this disease (Kasari et al., 2008; Kakani et al., 2010). To date, the susceptibility of native wildlife species inhabiting the US to RVFV has not been investigated and the potential role of these animals in RVF epidemiology remains widely unknown (Hartley et al., 2011). Knowing potential hosts of RVFV is important for determining the epidemiological risk and creating accurate prediction models for effective response and control strategies. In the current study, a variety of cell lines derived from native livestock and wildlife found throughout the US were tested for RVFV susceptibility and permissiveness. Initial experiments were performed with a vaccine strain of RVFV (MP-12) followed by comparison with a virulent wild type RVFV strain (SA01-1322) in representative cell lines. The MP-12 strain originated from the passage 12 attenuated ZH548 isolate from the serum of a febrile human case during the Egyptian RVFV outbreak in 1977 (Caplen et al., 1985). The MP-12 strain and several of its derivatives have since been researched and pursued as vaccine candidates in the US (Kortekaas, 2014). The SA01-1322 strain was originally isolated from a mosquito in Saudi Arabia during the 2000–2001 RVFV outbreak which resulted in more than 2000 human deaths and significant livestock deaths (Al-Hazmi et al., 2003; Madani et al., 2003). RVFV is listed as a category A high-priority pathogen by the National Institute of Allergy and Infectious Diseases and a select agent by the U.S. Department of Health and Human Services and the U.S. Department of Agriculture, and requires work to be performed at a biosafety level 3+ (BSL-3+) high biosecurity containment facility. The MP-12 strain is excluded from the select agent list and considered a BSL-2 agent in the US, which makes it a convenient model virus to work with. This work serves as a guide for future animal model and risk assessment studies concerning this important zoonotic pathogen.

## Materials and Methods

### Virus

The attenuated RVFV MP-12 strain was provided by the United States Army Medical Research Institute for Infectious Diseases. The virulent Saudi Arabia isolate SA01-1322 (Miller et al., 2002) was provided by R. Bowen, Colorado State University through B. Miller, Centers for Disease Control, Fort Collins, CO, USA. Experiments with the SA01-1322 strain were performed at Kansas State University’s Biosecurity Research Institute BSL-3+ laboratory, and the MP-12 experiments were completed outside of the high-containment facility at a BSL-2 laboratory. Both virus strains were propagated in mammalian-derived cell cultures and low passage stocks were used for experiments.

### Cell Lines

The African green monkey Vero cell line is a clone from the Middle America Research Unit (MARU). The frog (*Xenopus laevis*) kidney cell line was obtained from Jane Homan at the University of Wisconsin, and is commercially available through American Type Culture Collection (ATCC), Manassas, VA, USA. The sheep (*Ovis aries*, breed unknown), calf (*Bos taurus*, breed: black angus), pig (*Sus scrofa*, breed: duroc), American pronghorn (*Antilocapra americana*), elk (*Cervus elaphus*), mule deer (*Odocoileus hemionus*), white-tailed deer (*O. virginianus*), and coyote (*Canis latrans*) cell lines were initiated and established by the Arthropod-Borne Animal Diseases Research Unit (ABADRU), Manhattan, KS, USA from tissues derived from specimens donated to ABADRU or the Wyoming State Veterinary Diagnostic Laboratory. Briefly, primary lung and kidney cells were harvested by manual homogenization, and then pressed through tissue sieves. Subsequent cells were washed and resuspended in 199E media (Life Technologies, Grand Island, NY, USA) supplemented with 10% gamma-irradiated fetal bovine serum (FBS; Sigma–Aldrich, St. Louis, MO, USA) and 1x antibiotics (penicillin and streptomycin)-antimycotic (amphotericin B; PSF; Life Technologies, Grand Island, NY, USA). Non-adherent cells were removed and media replaced at weekly intervals. Cell lines were screened for bacteria, fungi, and mycoplasma contamination by cultivation methods or using the MycoTect kit (Life Technologies, Grand Island, NY, USA). Specific tests for the presence of bovine viral diarrhea and bluetongue viruses were also performed. Certain cell lines were additionally screened for other viral contaminants by electron microscopy using negative staining techniques. All of the cell lines used for this study were negative for contaminants included in the screening processes mentioned. Lung cultures were a mixture of fibroblastic and endothelial like cells. All brain cell lines used in this study were prepared similar to the lung and kidney cell lines as described above and then transformed. Transformation consisted of transfection of primary cultures with an expression plasmid carrying the simian virus 40 genome (pBRSV, ATCC 45019) using Lipofectamine 2000 reagent (Life Technologies, Grand Island, NY, USA) per the manufacturer’s instructions. Medium was changed 48 h later to OptiMEM (Life Technologies, Grand Island, NY, USA) and 10% FBS, and the cells were grown for an additional 1 to 2 weeks, when clusters of cells showing loss of contact inhibition and an increased rate of cell division were selected using cloning cylinders. All cell lines were maintained at 5% CO_2,_ 37°C with the exception of the frog cell line which was kept at 28–30°C.

### Viral Growth Kinetics

Overnight cell cultures (∼90% confluent) were infected at 0.1 multiplicity of infection (MOI) with either the MP-12 or SA01-1322 RVFV strains. Virus was adsorbed for 1 h then monolayers were washed and fresh media added. Time course samples were collected at 0, 6, 12, 24, 36, and 48 h post infection. Cells and culture supernatant were collected separately for intracellular and extracellular virus titration, respectively. Total virus was measured by collecting both cells and culture supernatant simultaneously. Samples were subjected to three freeze/thaw cycles and stored at -80°C until analysis. Standard plaque assays on Vero MARU cells were performed to calculate titers in plaque forming units per ml (pfu/ml).

### RNA Extraction and Real Time Reverse Transcription Polymerase Chain Reaction (rRT-PCR)

Total RNA was extracted from virus time course samples using TRIzol-LS reagent (Life Technologies, Grand Island, NY, USA) and the magnetic-bead based capture MagMAX viral RNA isolation kit (Life Technologies, Grand Island, NY, USA). Briefly, 100 μl of aqueous phase was added to 90 μl of isopropanol and 10 μl bead mix. Total sample RNA was washed four times and eluted in 30 μl of elution buffer by use of the automated KingFisher Magnetic Particle Processor (Thermo Fisher Scientific Inc., Waltham, MA, USA). Sequences of primers and probes directed toward a region of the large (L) genome segment of MP-12 or field strains of RVFV have been published previously and were used to estimate viral RNA levels (Bird et al., 2007; Wilson et al., 2013). Beta actin (Moniwa et al., 2007) was used as a reference gene to calculate the relative levels of viral RNA among samples using the comparative Ct method (Delta Delta Ct). The relative levels of extracellular viral RNA were calculated by comparison to the beta actin levels in the intracellular fraction of the same sample. Beta actin and MP-12 or RVFV primer and probe sets were ran as a duplex real time reverse transcription polymerase chain reaction (rRT-PCR) assay using 5 μl sample RNA template and the one-step AgPath ID rRT-PCR kit (Life Technologies, Grand Island, NY, USA) under conditions previously published (Wilson et al., 2013). The primer and probe sequences are listed in **Table 1** and locations of the primer and probes on the RVFV L segment are shown in the Supplementary Figure S1.

**Table 1:** Primer and probes for real time reverse transcription polymerase chain reaction (RT-PCR).

Primer/Probe	Position	Sequence 5′–3′	Reference
Rift Valley fever virus (RVFV)-L-forward	2912–2933	TGA-AAA-TTC-CTG-AGA-CAC-ATG-G	Bird et al. (2007)
RVFV-L-reverse	2981–3001	ACT-TCC-TTG-CAT-CAT-CTG-ATG	
RVFV-L-probe	2950–2977	(CAL Red)^a^ CAA-TGT-AAG-GGG-CCT-GTG-TGG-ACT-TGT-G (Iowa Black)^b^	
MP12-L-forward	3296–3316	CCT-CAC-TAT-TAC-ACA-CCA-TTC	Wilson et al. (2013)
MP12-L-reverse	3436–3453	ATC-ATC-AGC-TGG-GAA-GCT	
MP12-L-probe	3371–3385	(FAM)^a^ CTG-AGA-TGA-GCA-AGA (Iowa Black)^b^	
Beta actin-forward		BTC-CTT-CCT-GGG-CAT-GGA	Moniwa et al. (2007)
Beta actin-reverse		GRG-GSG-CGA-TGA-TCT-TGA-T	
Beta actin-probe		(CY5)^a^ TCC-ATC-ATG-AAG-TGY-GAC-GTS-GAC-A (Iowa Black)^b^	

*^a^Fluorescent reporter dye; ^b^Quencher*.

## Results

**FIGURE 1:**
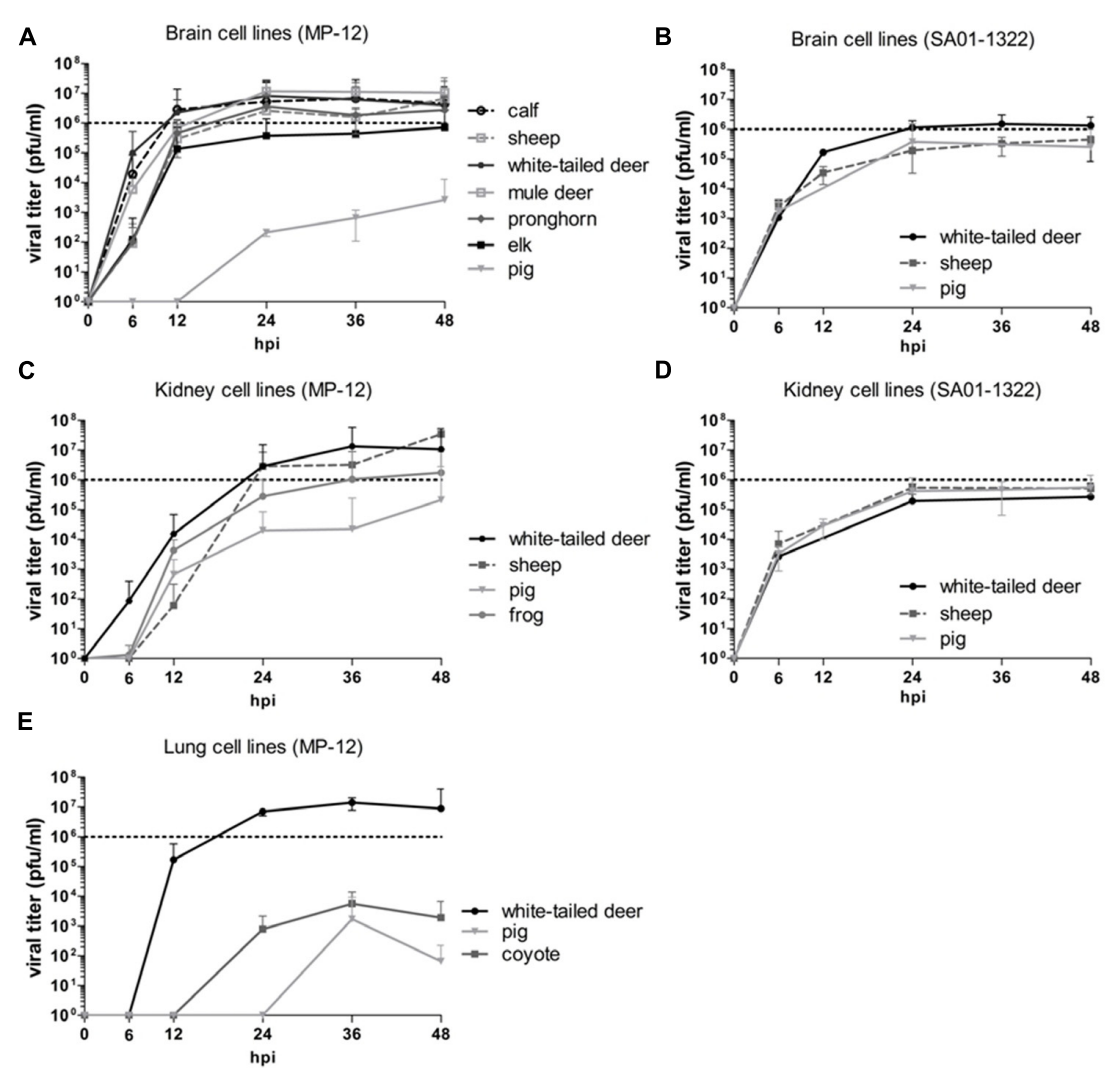
**Rift Valley fever virus (RVFV) replication kinetics in North American livestock and wildlife cell lines.** Cell cultures derived from brain **(A,B)**, kidney **(C,D)**, and lung **(E)** were infected with MP-12 **(A,C,E)** or SA01-1322 **(B,D)** strains at 0.1 multiplicity of infection (MOI) and the viral titers were calculated by standard plaque assay at the indicated hours post infection (hpi). The mean of at least three biological replicates are represented with 95% confidence intervals.

A total of nine different animal-derived cell lines indigenous to North America were evaluated, including domestic ruminant (sheep and calf), wild ruminant (white-tailed deer, mule deer, pronghorn, elk), and non-ruminant (pig, coyote, frog) species. In both animals and humans, RVFV typically targets the liver and spleen, and can be neuroinvasive; however, it is also known to replicate in a variety of other tissues and cells (Ikegami and Makino, 2011). Cell lines derived from kidney, brain, and lung tissues were compared due to the availability of these tissues across multiple animal species. The attenuated RVFV MP-12 strain was used for initial comparisons of virus replication in various cell lines, as it does not require work at a high biosecurity level facility. Viral growth kinetics were then performed with a representative set of cell lines with the virulent SA01-1322 strain RVFV. The brain cell lines from domestic and wild ruminants supported the most efficient viral replication of the MP-12 strain. Using a MOI of 0.1, viral titers in the ruminant-derived brain cell lines peaked around 24 hpi and MP-12 titers ranged between 10^5^ and 10^7^ pfu/ml (**Figure 1A**). Replication kinetics of MP-12 were more rapid in the calf, white-tailed, and mule deer cell lines, with titers nearly 10^2^ to 10^3^ pfu/ml higher at 6 hpi compared to titers from sheep, pronghorn, and elk cell cultures (**Figure 1A**). Replication kinetics of MP-12 in the pig brain cell line was significantly delayed compared to the ruminant brain cells. Expansion of MP-12 in the pig brain cells did not occur until after 12 hpi and only a titer around 10^3^ pfu/ml was achieved by 48 hpi which was more than 10^2^ pfu/ml lower than the lowest titer observed at the same time point among the ruminant-derived brain cell lines (**Figure 1A**). Conversely, replication kinetics and titers of the SA01-1322 strain were more consistent among the white-tailed deer, sheep, and pig brain cell lines tested (**Figure 1B**). Titers averaged around 10^3^ pfu/ml by 6 hpi and then peaked around 10^5^ to 10^6^ pfu/ml between 24 and 36 hpi, with the white-tailed deer cell line supporting slightly higher virus titers (**Figure 1B**).

The replication kinetics of the MP-12 strain were slightly slower in the kidney cell lines compared to the brain cell lines with titers peaking after 36–48 hpi (**Figure 1C**); however, high titers were still achieved in these cell lines. Titers of MP-12 in the white-tailed deer and sheep kidney cell lines reached 10^7^ pfu/ml, while the pig and frog kidney cell lines supported MP-12 titers of 10^5^ and 10^6^ pfu/ml, respectively (**Figure 1C**). Similar replication kinetics and titers in the range of 10^5^ pfu/ml were observed for the SA01-1322 strain among the white-tailed deer, sheep, and pig kidney cell lines (**Figure 1D**). Compared to the brain cell line, SA01-1322 peak titers were about 10^1^ pfu/ml lower in white-tailed deer kidney cells (**Figures 1B,D**).

Although RVFV is not typically associated with respiratory disease, results from this study demonstrated that MP-12 was capable of replicating in three different lung cell lines. Lung-derived cell lines consisted of a heterogeneous mixture of cells showing epithelial and fibroblast-like morphology. Titers in the lung cells peaked around 10^3^ pfu/ml in pig and coyote cells, and 10^7^ pfu/ml in white-tailed deer cells by 36 hpi (**Figure 1E**). Infectious virus was detected by 12 hpi in the white-tailed deer lung cells and by 24 hpi in the coyote lung cell line (**Figure 1E**). The pig lung cell line had the most delayed viral kinetics with virus not detected until 36 hpi (**Figure 1E**). Overall, the SA01-1322 strain viral kinetics and titers were relatively consistent with little variation among the cell lines tested, while the MP-12 strain exhibited more variability among species and the tissues the cell lines were derived.

**FIGURE 2:**
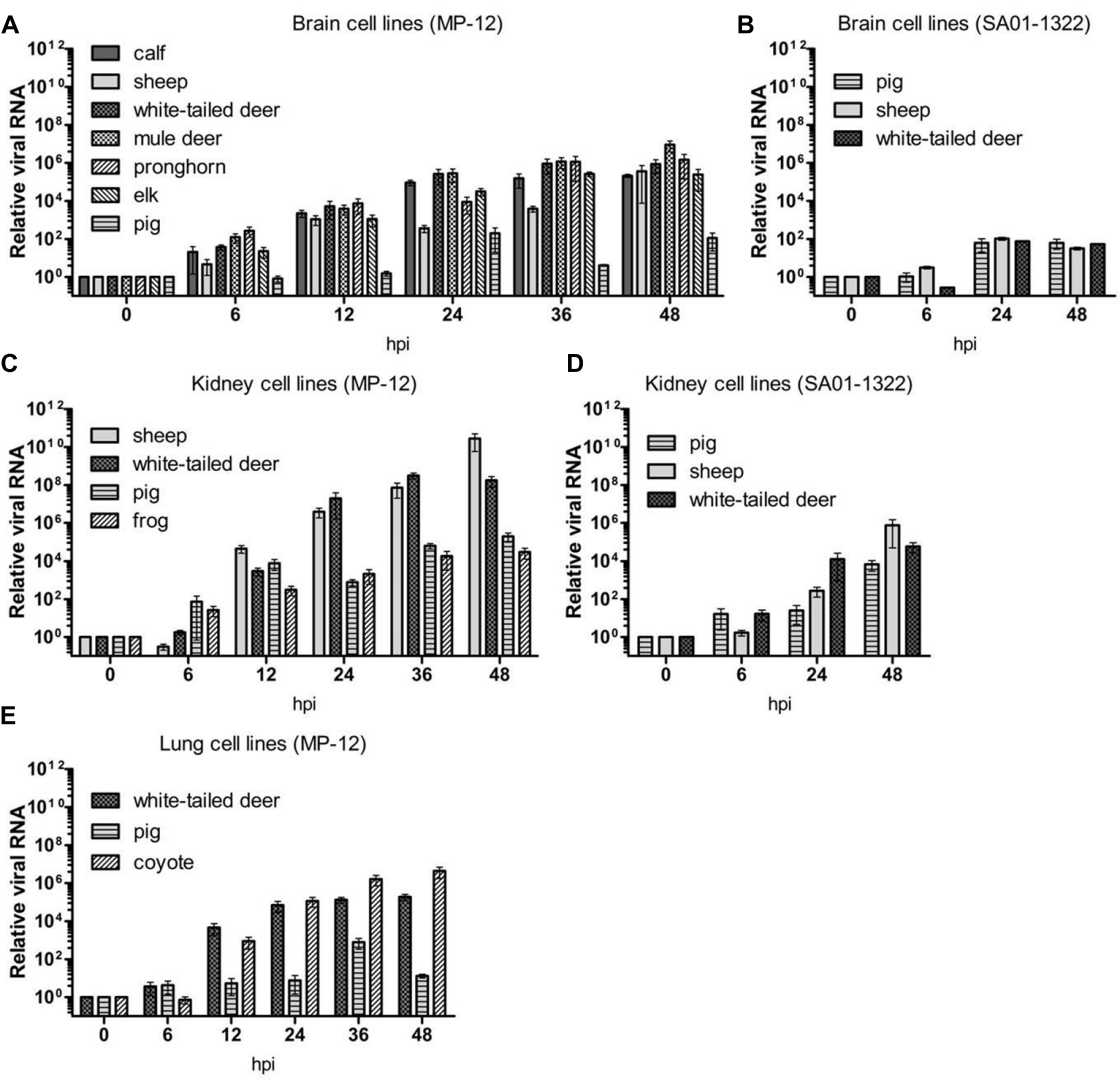
**Relative viral RNA levels in North American livestock and wildlife cell lines.** Real time reverse transcription polymerase chain reaction (RT-PCR) was performed on time point samples from brain **(A,B)**, kidney **(C,D)**, and lung **(E)** cell lines infected with MP-12 **(A,C,E)** or SA01-1322 **(B,D)** strains. Expression of viral RNA was calculated relative to beta actin using the comparative Ct method. The mean of at least three biological replicates are represented with SEs.

The L genome segment of RVFV was targeted to monitor relative viral RNA levels in the time course samples. In general, relative viral RNA levels correlated with the kinetics of virus titers; however, the amount of viral RNA did not always directly correspond to the infectious viral titer (**Figures 1** and **2**). For example, SA01-1322 RNA levels in the brain cell lines (**Figure 2B**) were lower than SA01-1322 RNA in kidney cells (**Figure 2D**). Levels of MP-12 RNA were generally higher than the RNA levels produced by the SA01-1322 strain (**Figures 2A–D**). This observation could be explained by more efficient primer binding and amplification of the MP-12 RNA template compared to the SA01-1322 strain, although no mutations were detected in the primer or probe binding sites by sequencing of the original virus stock used in these experiments (Supplementary Figure S1). Alternatively, this could be due to efficient expression, but less efficient packaging of infectious virus particles, or more defective interfering particles incorporated in the MP-12 compared to the SA01-1322 virus strain. Based on these results, the amount of viral RNA produced in cells determined by rRT-PCR does not accurately correlate to infectious virus titer, but can be useful for monitoring viral replication kinetics and perhaps estimating infectious titer for a defined viral isolate.

**FIGURE 3:**
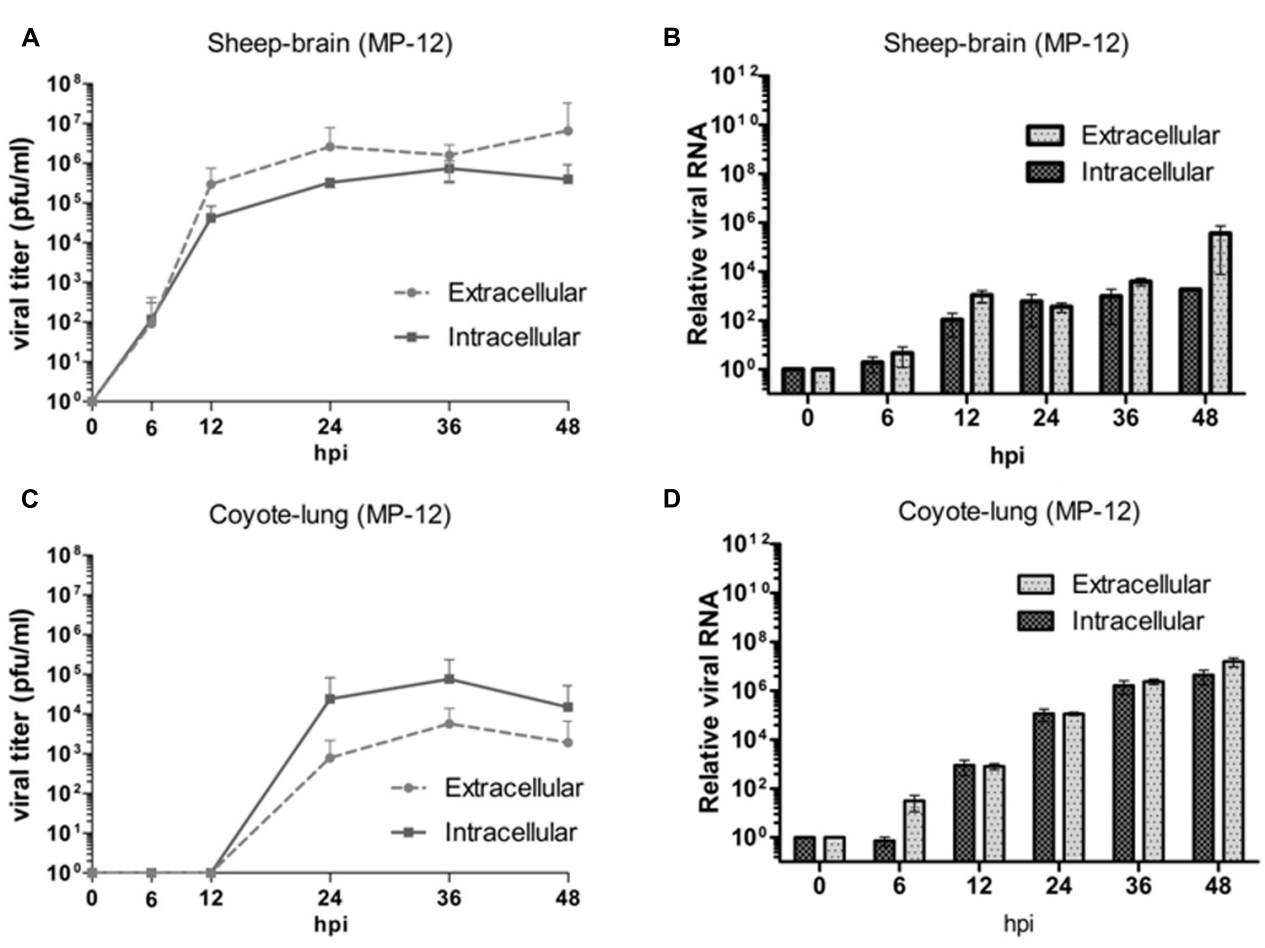
**Comparison of intracellular and extracellular virus in North American livestock and wildlife cell lines.** Intracellular and extracellular virus titers **(A,C)** and relative viral RNA levels **(B,D)** are shown. The mean of at least three biological replicates are represented.

In order to determine the ability of RVFV to be efficiently released from infected cells, extracellular virus from the culture supernatants and intracellular virus from cells was also compared. Although not statistically significant, there were some observed differences between the two fractions from certain cell lines that were evaluated. In particular, the brain cell lines derived from sheep, calf, and white-tailed deer which supported efficient viral replication had slightly higher extracellular virus titers compared to intracellular (**Figure 3A** and data not shown), while the inverse was observed in cell lines with more delayed viral kinetics such as the coyote lung and pig kidney cell lines (**Figure 3C** and data not shown). The difference between intra- and extracellular viral titers was not as clearly observed with the viral RNA levels (**Figures 3B,D**). While lower MP-12 titers were observed in the coyote cell line compared to the sheep cells (**Figures 3A,C**), the viral RNA was higher in the coyote cells than the sheep cells (**Figures 3B,D**).

## Discussion

Domestic ruminants are the primary hosts for RVFV (Pepin et al., 2010); however, there is evidence of wild ruminant species in endemic regions also being infected including buffalo, waterbuck, impala, and kudu (Evans et al., 2008; LaBeaud et al., 2011; Britch et al., 2013). There is an abundance of similar wild ruminants, such as the American pronghorn, elk, and multiple deer species, widely distributed throughout the US. The overlap of wildlife habitats with livestock and human populations increases the risk for transmission and dissemination of zoonotic arboviruses like RVFV. Specifically, white-tailed deer are a common source of mosquito blood meals (Apperson et al., 2004; Molaei et al., 2008, 2009a,b), and serve as reservoirs for other arboviruses such as Jamestown Canyon virus which affects humans, Cache Valley, and epizootic hemorrhagic disease viruses of sheep and cattle (Molaei et al., 2009a; Allison et al., 2010). Results from the current study demonstrated that wild ruminant-derived cell lines, especially white-tailed deer, supported efficient replication of two different strains of RVFV to high titers. This could have a significant impact on RVFV ecology as well as on the deer farming industry. Thus, these wild ruminants should be considered as high priority candidates for further investigation including competency and transmission studies. Controlled infection studies with white-tailed deer have been accomplished with other viruses, and plans for a RVFV infection study are currently in progress.

Seroprevalence studies have shown RVFV exposure of a number of non-ruminant animals. Evidence in Egypt found seroprevalence in domestic pigs, and also in the desert warthog (Evans et al., 2008). Experimentally, pigs appear to be clinically resistant (Easterday and Murphy, 1963; Easterday, 1965; Shimshony and Barzilai, 1983), while others have shown that resistance is dose-dependent (Scott, 1963). Feral swine are often of epidemiological concern because they are carriers of a variety of parasites and microbial diseases which could potentially threaten the health of domestic livestock, wildlife, and humans (Mayer and Brisbin, 1991). Furthermore, their numbers and distribution continue to grow in the US (Mayer and Brisbin, 1991). Swine are not known to play a significant role in RVFV epidemiology in endemic countries (Olaleye et al., 1996); however, infection of pigs could have significant economic repercussions related to pork production and trade. Although replication of the MP-12 strain was the most inhibited in the pig cell lines, the virulent SA01-1322 RVFV strain replicated nearly as efficiently in the pig cell lines as in the sheep cell lines tested. Therefore, further investigation and assessment of swine for RVFV susceptibility may be warranted.

Some experimental evidence exists that other non-ruminant species such as domestic horses, dogs, and cats may be mildly susceptible to RVFV in endemic countries, while aves, reptiles, and amphibians appear to be refractory (Olive et al., 2012). We tested a coyote cell line since the distribution of these animals’ spans throughout North America and it is possible that coyotes could become exposed by scavenging infected livestock or wildlife, or by infected mosquitoes. Although less efficiently than in the other cell lines tested, the MP-12 strain did replicate in the coyote-derived lung cell lines. Unfortunately, other tissues from coyote were not available for testing. Interestingly, the frog kidney cell line was also found to support RVFV replication. Wetland areas contain a variety of amphibians and reptiles, and are ideal breeding grounds for mosquitoes known to feed on them (Burkett-Cadena et al., 2008; Bingham et al., 2014). An enzootic cycle has recently been demonstrated between mosquitoes and snakes for eastern equine encephalitis virus (White et al., 2011; Bingham et al., 2012; Graham et al., 2012). In an analysis of blood meal sources of mosquitoes collected from Kenya during the 2006–2007 RVFV outbreak, 13% of one of the predominant infected mosquito species was found to have fed on frogs (Lutomiah et al., 2014). Therefore, it may be important not to completely rule out these animals as playing a potential role in the viral life cycle of RVFV. In summary, it may be necessary to consider a wide variety of animals when assessing the risk of infection, dissemination, and maintenance of this important zoonotic arbovirus. Although especially challenging under high biosecurity conditions, controlled experimental animal studies with wildlife species such as white-tailed deer and coyotes have been achieved with other pathogen models, and are therefore not out of the realm of possibility to pursue further.

Finally, it is interesting to note that three different lung cell lines were permissive to RVFV infection, even though RVFVis is typically not associated with respiratory disease. Infection by aerosol transmission of RVFV has occurred in the laboratory environment; however, no human-to-human transmission has ever been documented (CDC, 2013). Intranasal challenge and aerosol exposure models with RVFV in mice and monkeys results in severe neuropathology and death (Bales et al., 2012; Reed et al., 2013; Hartman et al., 2014). In a recent sheep model challenged subcutaneously, coughing was observed and low levels of RVFV (less than 10^2^ pfu/mL) were detected in nasal swabs from some of the infected animals; however, no signs of viremia were detected from any of the uninfected control animals housed alongside the challenged animals throughout the 21 days study (unpublished data). Although it may be possible for airborne transmission to occur, it is more likely to occur through direct contact with infected tissues and bodily fluids. Nonetheless, creation and exposure to aerosols of RVFV should be avoided due to the severe pathology associated with this route of infection. Further investigation is necessary for understanding better the potential risk of aerosol transmission of RVFV.

## Conclusion

The objective of the current study was to build a foundation for future *in vivo* host competency studies and guide statistical risk modeling for this high-consequence zoonotic pathogen. The results presented here suggest RVFV could potentially infect a wide range of domestic livestock and wildlife native to North America. All of the cell lines tested in the current study were permissive and susceptible for RVFV replication, especially ruminant-derived cell lines. Based on these results and serology surveys of wild ruminants in endemic countries, deer and other wild ruminant species should be given high priority for further investigations. It is currently unknown whether these animals are competent hosts for RVFV, or what role they could play in the dissemination of this virus. In the event that RVFV were introduced to the US and wildlife was capable of becoming reservoirs for this pathogen, controlling the spread of RVFV would be difficult, if not impossible. Not only is RVFV a threat to both human and animal health, but the establishment of this pathogen would have costly economic impact related to livestock production and trade. Thus, expanding research to investigate competence of potential wildlife hosts in the US remains critical in order to plan effective response and control strategies if an introduction were to occur.

## Author Contributions

NG, SI, and PB contributed to the acquisition of data. NG, JR, and WW contributed to experimental design, and the analysis and interpretation of data. All authors contributed to the drafting and final approval of the manuscript for submission, and take responsibility for the work presented.

## Conflict of Interest Statement

The authors declare that the research was conducted in the absence of any commercial or financial relationships that could be construed as a potential conflict of interest.
