# Supplementary figures and images for: Comparison of Rift Valley fever virus replication in North American livestock and wildlife cell lines
Source: Front Microbiol. 2015 Jun 30;6:664. doi: 10.3389/fmicb.2015.00664 (PMC4485352; doi:10.3389/fmicb.2015.00664)

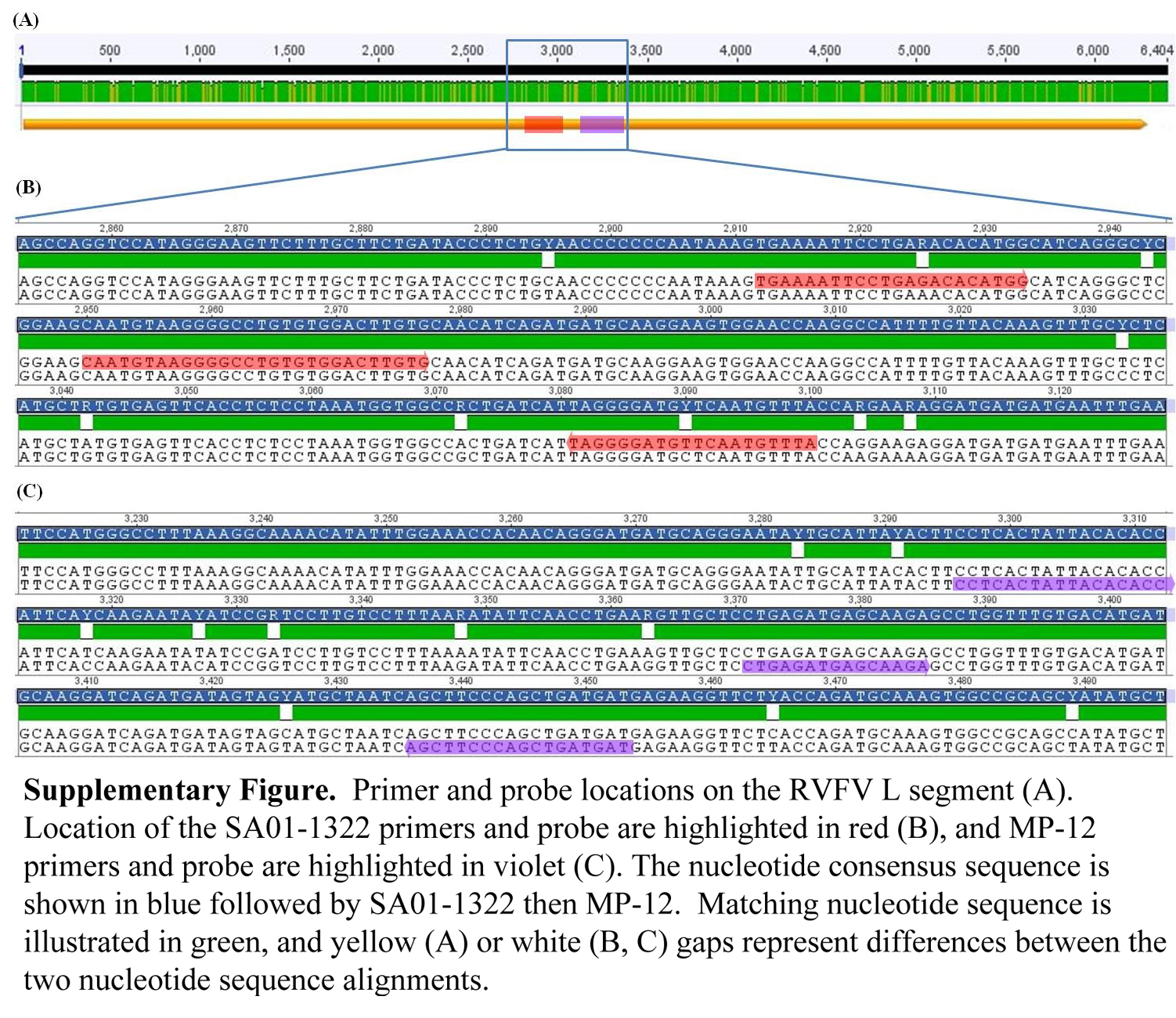

Supplement: Supplementary file 1 [file Image_1.JPEG]
